# Hypoxia-Induced MicroRNA-212/132 Alter Blood-Brain Barrier Integrity Through Inhibition of Tight Junction-Associated Proteins in Human and Mouse Brain Microvascular Endothelial Cells

**DOI:** 10.1007/s12975-018-0683-2

**Published:** 2019-01-08

**Authors:** Malgorzata Burek, Anna König, Mareike Lang, Jan Fiedler, Sabrina Oerter, Norbert Roewer, Michael Bohnert, Serge C. Thal, Kinga G. Blecharz-Lang, Johannes Woitzik, Thomas Thum, Carola Y. Förster

**Affiliations:** 1 Department of Anesthesia and Critical Care, University of Würzburg, Würzburg, Germany; 2 Hannover Medical School, Institute of Molecular and Translational Therapeutic Strategies (IMTTS), Hannover, Germany; 3 Institute of Forensic Medicine, University of Würzburg, Würzburg, Germany; 4 Department of Anesthesiology, Medical Center of the Johannes Gutenberg-University Mainz, Mainz, Germany; 5 Department of Experimental Neurosurgery, Charité – Universitätsmedizin Berlin, Corporate Member of Freie Universität Berlin, Humboldt-Universität zu Berlin and Berlin Institute of Health, Berlin, Germany; 6 Department of Neurosurgery and Center for Stroke Research Berlin (CSB), Charité – Universitätsmedizin Berlin, Berlin, Germany

**Keywords:** MicroRNA-212/132, Blood-brain barrier, Tight junctions, Oxygen-glucose deprivation, Traumatic brain injury, Stroke

## Abstract

Blood-brain barrier (BBB) integrity is one of the important elements of central nervous system (CNS) homeostasis. MicroRNAs (miRs) have been demonstrated to play a role in many CNS disorders such as stroke and traumatic brain injury. MiR-212/132 are highly expressed in the CNS but their role at the BBB has not been characterized yet. Thus, we analyzed the expression of miR-212/132 in hypoxic mouse and human brain microvascular endothelial cells (BMEC) as well as in posttraumatic mouse and human brain tissue and serum exosomes. MiR-212/132 expression was detected in brain capillaries by in situ hybridization and was increased up to ten times in hypoxic BMEC. Over-expression of pre-miR-212/132 in BMEC decreased barrier properties and reduced migration of BMEC in the wound healing assay. We identified and validated tight junction proteins claudin-1 (Cldn1), junctional adhesion molecule 3 (Jam3), and tight junction-associated protein 1 (Tjap1) as potential miR-212/132 targets. Over-expression of miRs led to a decrease in mRNA and protein expression of Cldn1, Jam3, and Tjap1, which could be rescued by a respective anti-miR. In conclusion, our study identifies miR-212/132 as critical players at the hypoxic BBB. In addition, we propose three new direct miR-212/132 targets to be involved in miR-212/132-mediated effects on BBB properties.

**Electronic supplementary material:**

The online version of this article (10.1007/s12975-018-0683-2) contains supplementary material, which is available to authorized users.

## Introduction

Hypoxic brain microvascular endothelial cells respond strongly to the hypoxic trigger and nutrient deprivation by induction of multiple signaling pathways at the blood-brain barrier (BBB) leading to functional changes in the neurovascular unit (NVU) [1–3]. Such hypoxic conditions at the NVU are observed after ischemic stroke or traumatic brain injury (TBI). Both conditions induce BBB damage, production of reactive oxygen species, excitotoxicity, post-ischemic brain inflammation, and neuronal cell death [4]. Despite extensive research efforts, there is still no successful therapy for stroke and TBI, besides treatments aiming at symptomatic removal of the occlusion and stimulation of self-healing processes in the brain [5]. MicroRNAs (miRs) are single stranded RNA molecules of about 22 nucleotides that regulate gene expression by binding to the 3′-untranslated region (3′-UTR) of specific target mRNAs [6]. MiRs have been shown to be induced in stroke and TBI and could therefore bear a therapeutic potential [7, 8]. MiR-132 and miR-212, which belong to the same family, are located on chromosome 17 in humans and on chromosome 11 in mice. MiR-132 and miR-212 are highly conserved between species and share common target genes [9]. Sequences of mouse and human miR-132 are identical, while mouse miR-212 has one additional nucleotide at the 3′-end [10]. MiR-212/132 are highly expressed in the CNS where they play a role in synaptic plasticity, memory formation, and neurite outgrowth [10]. On the other hand, miR-212/132 seem to be of significance in cardiac hypertrophy and in endothelial cell function [11, 12]. MiR-212/132 are downregulated in Alzheimer’s disease and miR-212/132 knockout mice show memory deficits as well as increased Tau-protein levels in the brain tissue [13–15]. The role of miR-212/132 has not been characterized yet in brain vascular endothelium and BBB function.

In this study, we found that miR-212/132 are induced in mouse and human hypoxic BBB. In addition, we uncovered a novel role for miR-212/132 as regulators of hypoxic BBB function by targeting the transcription of tight junction and tight junction-associated proteins. We identified and validated three new targets of miR-212/132, which play a role in the maintenance of BBB integrity. Specific manipulation of miR-212/132 levels at the BBB could be used to change the barrier properties of the BBB, e.g., in order to deliver therapeutic molecules into the brain.

## Material and Methods

### Cell Culture and Transfection

Mouse microvascular cerebral (cEND) endothelial cell line was isolated, immortalized, and cultivated as described previously [16–18]. Briefly, brains from 3 to 5 days old mice (strain 129Sv) of either sex were minced in buffer A (15 mM HEPES, pH 7.4; 153 mM NaCl; 5.6 mM KCl; 2.3 mM CaCl_2_ × 2 H_2_O; 2.6 mM MgCl_2_ × 6 H_2_O; 1% BSA). Brain tissue was digested with 0.75% (*w*/*v*) collagenase A (Roche) for 30 min at 37 °C. The suspension was diluted 1:10 with buffer A and centrifuged through a 25% (*w*/*v*) BSA gradient for 20 min at 1000×*g*. Cell pellet was washed with buffer A and plated on 24-well plates coated with collagen IV in DMEM with 10% FCS. Immortalization of endothelial cells was achieved by transfection with Polyoma middle T antigen. The cells showed stable properties up to 50 passages. Primary mouse brain microvascular endothelial cells (PELOBiotech) were cultured in ECM Medium (PELOBiotech) according to the manufacturer’s recommendations and were used up to five passages [19]. Human microvascular cerebral endothelial cell line hDMEC/D3 was cultured as previously described and was used at passage 45 [20, 21]. Rat glioma cell cline C6 was obtained from ATCC and cultured in cEND medium. Endothelial cell lines were grown on plates coated with collagen IV (Fluka). For oxygen-glucose deprivation (OGD) treatment, a C6 glioma-conditioned medium was prepared. First, C6 glioma cells were subjected to OGD in glucose-free medium and 1% O_2_ for 4 h. Hypoxic C6 glioma medium was collected and mixed in proportion 1:1 with fresh glucose-free medium. This C6 glioma-conditioned medium was used for OGD experiments with cEND cells. CEND were subjected to OGD for 4 h and were either directly harvested or subjected to re-oxygenation under normal culture conditions for another 20 h. For transfection, cEND were grown to confluence. Over-expression of miRs was achieved by transfection with 500 ng miRNASelect™ pEGP-mmu-mir-132 and pEGP-mmu-mir-212 expression vectors (Cell Biolabs, Inc.) or respective pre-miRs. Pre-miRs hsa-miR-132-3p, mmu-miR-212-3p, and anti-miRs with corresponding scrambled controls (20 nM each) (Thermo Fisher Scientific) were transfected into the cells using RNAiFect Transfection Reagent (Qiagen) according to manufacturer’s instructions.

### Traumatic Brain Injury

Brain tissue from mice subjected to brain trauma by controlled cortical impact (CCI) was provided by Dr. Thal (University of Mainz). The Animal Ethics Committee of the Landesuntersuchungsamt Rheinland-Pfalz approved the mice experiments (protocol number 23177-07/G10-1-024) and the experiments were performed in compliance with the institutional guidelines of the Johannes Gutenberg University, Mainz and in accordance with the German law for animal protection. The data are reported according to ARRIVE (Animal Research Reporting in Vivo Experiments) guidelines. Twelve 8–10-week-old male C57BL/6 mice (Charles River Laboratories) were randomly assigned to sham and TBI group and experimental procedures were performed as described previously [3]. The animals were anesthetized using isoflurane in an air mixture (40% O_2_ and 60% N_2_). A pneumatic brain trauma was performed on the right parietal cortex on dura-covered brain via CCI (1.5 mm displacement). After injury, mice recovered in their individual cages for 2 h in a humidity- and temperature-controlled incubator (IC8000, Draeger). Animals were sacrificed 24 h post CCI, brain were collected and frozen for further analysis.

### Human Brain Tissue

Human TBI and control brain tissue samples were provided by the Institute of Forensic Medicine (University of Würzburg). Experiments with samples taken from deceased that underwent medico-legal autopsy at the Institute of Forensic Medicine were approved by the ethical committee of the Julius-Maximilians-University Würzburg. Hereby we followed and strictly adhered to the Ethical Guidelines of the University of Würzburg, which are in accordance with Helsinki Declaration of 1975 and its revision of 1983.

### Isolation of Exosomes from Serum of Patients with Malignant Hemispheric Stroke

Serum collection from stroke patients was approved by the local research and Ethics Commission of the Charitè-Universitätsmedizin Berlin (reference EA4/118/13). Informed consent was obtained from all individual participant or the legal representative. The Ethical Guidelines of the Charitè, which are in accordance with the Helsinki Declaration of 1975 and its revision of 1983 were strictly followed. Serum from 14 patients with malignant hemispheric stroke (MHS) defined as subtotal or total middle cerebral artery infarction with or without additional infarction of the anterior or posterior cerebral artery and the clinical indication for decompressive hemicraniectomy was collected at day 3 and day 7 post stroke [22]. Serum samples have been used for isolation of serum exosomes and estimation of miR-212/132 expression level by qPCR. Serum exosomes were isolated from 50 μl of serum using Total Exosome Isolation (from serum) Kit (Thermo Fisher Scientific) according to the manufacturer’s instructions. Briefly, serum samples were centrifuged at 2000×*g* for 30 min to remove cells and debris. The clarified serum was transferred to a new tube and was mixed with 10 μl (0.2 volumes) of the Total Exosome Isolation (from serum) reagent. After mixing and incubating the sample at 4 °C for 30 min, exosomes were pelleted by centrifugation at 10,000×*g* for 10 min at room temperature. The supernatant was discarded and the exosomes were resuspended in 200 μl of exosome elution buffer. Total RNA containing microRNA fraction was isolated from exosomes using Total Exosome RNA and Protein Isolation Kit (Thermo Fisher Scientific) according to the manufacturer’s protocol. A synthetic cel-lin-4-5p mimic (Thermo Fisher Scientific) (25 fmol) was added as a spike-in control before RNA isolation. Total exosomal RNA was eluted with 50 μl of nuclease-free water. Two microliters of isolated RNA was used for cDNA synthesis with TaqMan® Advanced miRNA cDNA Synthesis Kit (Thermo Fisher Scientific). TaqMan Advanced miRNA Assays hsa-miR-212-3p, hsa-miR-132-3p, and cel-lin-4-5p were used with TaqMan Fast Advanced Master Mix (Thermo Fisher Scientific) in qPCR. Cel-lin-4-5p was used for normalization and calculation of relative expression by comparative CT method.

### qPCR

Capillaries were isolated from control and injured brain tissue as previously described [19]. Total RNA including small RNA fraction was isolated from cells or capillaries using *mir*Vana TM miRNA Isolation Kit (Thermo Fisher Scientific). RNA (1 μg) was subjected to reverse transcription with TaqMan Micro RNA Reverse Transcription kit (Thermo Fisher Scientific). TaqMan MicroRNA assay for mature miR-132 and miR-212 was used with TaqMan 2× Universal PCR Master Mix (Thermo Fisher Scientific) in StepOnePlus Real-Time PCR System (Thermo Fisher Scientific). U6 RNA and snoRNA55 were used for normalization. Expression analysis of miR-212/132 target genes was performed on 1 μg RNA which was transcribed using High Capacity cDNA Reverse Transcription Kit (Thermo Fisher Scientific). Commercially available TaqMan Gene Expression Assays (Thermo Fisher Scientific) and TaqMan Universal Master Mix II were used in qPCR reactions. β-Actin and 18S rRNA served as endogenous controls [23]. Relative expression was calculated by comparative CT method.

### In Situ Hybridization and Immunohistochemistry

Mouse brain cryosections (10 μm) were dried at room temperature, fixed with 4% paraformaldehyde (PFA) for 10 min, and washed two times with DEPC-PBS. The sections were treated with 2 μg/ml proteinase K (Roche) for 15 min at 37 °C and washed three times with DEPC-PBS. Then, the sections were acetylated 15 min at room temperature (acetic anhydride in DEPC-water, 6 N HCL, Triethanolamine) and subsequently washed three times with DEPC-PBS. Sections were prehybridized in hybridization buffer (50% formamide; 5× saline sodium citrate; pH 7.0; 100 μg/ml sheared salmon sperm DNA, 0.5 mg/ml yeast tRNA, 1× Denhardt’s solution) at 58 °C for 1 h before the buffer was replaced with hybridization solution containing miR probe. The miRCURY LNA microRNA detection probes labeled with fluorescein at 5′-end, scramble-miR (#99004-04), hsa-miR-132 (#38031-04), and mmu-miR-212 (#39550-04) were from Exiqon. Probes were diluted in pre-hybridization buffer to a concentration of 5 nM and hybridized with the sections overnight at 58 °C according to RNA melting temperature of probes. After hybridization, the sections were washed three times with 2× SSC and 0.2× SSC, permeabilized for immunostaining with 0.1% Triton X-100 and washed two times with PBS. Unspecific background was blocked with 5% swine serum diluted in PBS/BSA for 30 min. To stain endothelial cells, the sections were incubated overnight with the rat anti-Pecam-1 (CD31) (1:100; BD Biosciences) antibody at 4 °C. Then, sections were incubated with donkey anti-rat-Alexa 594 (1:400, Thermo Fisher Scientific) antibody for 1 h at room temperature. The sections were washed three times with PBS and were mounted with VectaShield (Vector Laboratories). Images were generated with a Keyence BZ9000 microscope (Keyence).

### Western Blot

Cells were lysed in ice-cold RIPA buffer containing a protease inhibitors cocktail (Roche). Protein content was quantified by BCA protein Assay Kit (Thermo Fisher Scientific). Of protein, 20 μg was subjected to electrophoresis and was transferred to Hybond nitrocellulose membranes (Promega) and blocked with 5% non-fat dry milk in Tris-buffered saline containing 0.05% Tween 20. Membranes were incubated overnight with primary antibodies respectively: anti-Jam-C (dilution 1:1000, Millipore), anti-claudin-1 (1:200, Thermo Fisher Scientific), anti-occludin (1:200, Thermo Fisher Scientific), and anti-Tjap1 (1:1000, Abcam). Monoclonal anti-β-actin antibody (1:5000, Sigma) was used as endogenous control. The specific bands were visualized using secondary anti-rabbit antibody (1:3000, GE Healthcare), Enhanced Chemiluminescence detection kit (Promega), and FluorChem FC2 Multi-Imager II (Alpha Innotech). Intensity of proteins bands was measured with ImageJ software.

### Measurements of Transendothelial Electrical Resistance and Permeability

For transendothelial electrical resistance (TEER) and permeability measurements, cEND cells were grown to confluence on transwells (0.4 μm pores, Greiner) coated with collagen IV followed by transfection as described above. TEER measurements were performed as previously described with chopstick electrode (Millipore) 24 h post-transfection [17, 24]. TEER values of blank filters were subtracted before calculating the final values. For permeability measurements, cell culture medium containing fluorescein (332 Da), FITC-Dextran 4 kDa, and FITC-Dextran 10 kDa (10 μM) was added to the upper chamber. After 2-h incubation at 37 °C, samples were collected from the bottom chamber and fluorescence was measured in triplicates using microplate reader (Tecan) at 485/535 nm. Values were presented as fraction of initial amount (%).

### Wound Healing Assay

The wound healing assay was performed as previously described [25]. Briefly, cEND were grown in μ-Dishes (Ibidi) in reservoirs separated by a 500-μm cell-free area to confluence. Cells were transfected as described above. After 24 h post-transfection, the silicone inserts were removed and the images corresponding to 0 h time-point were taken. The cells were allowed to grow for 48 h followed by microscopic documentation with Keyence BZ9000 microscope (Keyence). The cell-free area was calculated with Keyence software and was presented in arbitrary units.

### Luciferase Reporter Assay

The microRNA databases miRBase (http://microrna.sanger.ac.uk), PicTar (http://pctar.mdc-berilin.de), and TargetScan (http://www.targetscan.org) were used to identify potential miR-212/132 targets with known expression at the BBB. Predicted targets, the TJ and TJ-associated proteins Cldn1, Jam3, Ocln, and Tjap1 were chosen for further analysis. In order to validate the interaction between miRNAs and target genes mRNA, we applied a luciferase reporter assay as described previously [26–28]. Briefly, 3′-UTRs with putative miRNA binding sequences of Cldn1, Jam3, Ocln, and Tjap1 genes were cloned into the pMIR-REPORT vector (Promega). The resulting constructs (20 ng) were co-transfected with scr-miR or miR-212/132 (each 100 nM) and 20 ng of β-galactosidase control plasmid (Promega) into 48-well-plated HEK293 reporter cells by the use of Lipofectamine 2000 (Thermo Fisher Scientific). Cells were incubated for 24 h before detecting the luciferase and β-galactosidase activity applying respective substrates (Promega).

### Statistical Analysis

Data are expressed as mean ± SD. For statistical comparison of two groups, two-sided Student’s *t* test with same variances was used. For the comparison of three or more groups, we used one-way ANOVA followed by Sidak’s or Tukey’s multiple comparisons test (SigmaStat 3.1 and GraphPad Prism 7.00 Software). *P* values less than 0.05 were considered significant.

## Results

### Hypoxia Induces the Expression of miR-132 and miR-212 in Mouse and Human Brain Microvascular Endothelial Cells

**Fig. 1:**
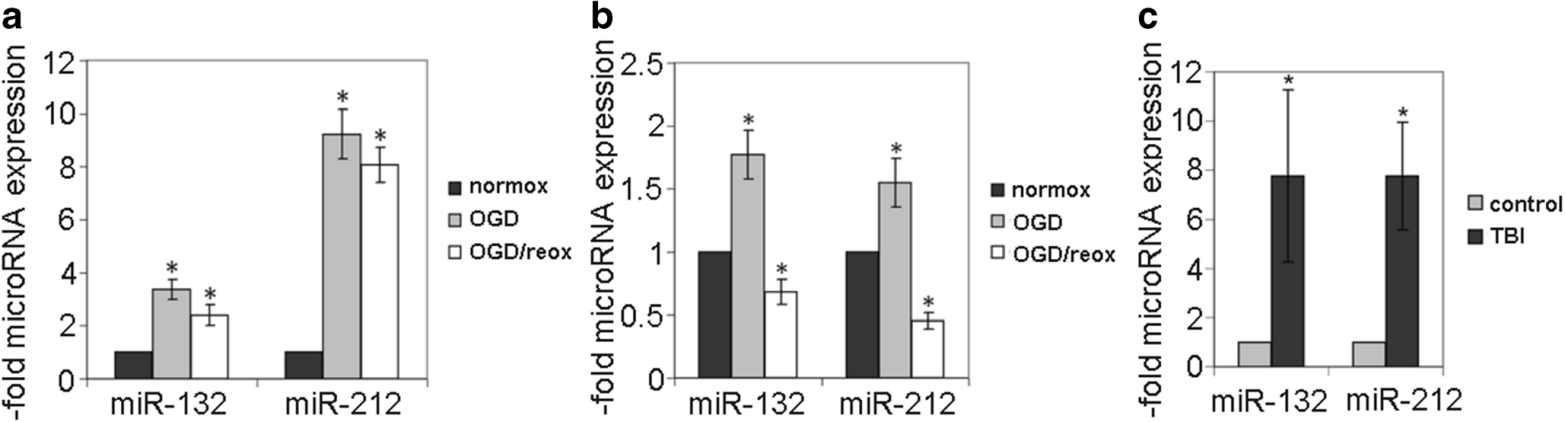
MiR-212/132 are upregulated after oxygen-glucose deprivation in mouse brain microvascular endothelial cells. MiR-212/132 levels were measured in oxygen-glucose deprivation (OGD)- or OGD/re-oxygenation (OGD/reox)-treated cEND cells (**a**) and mouse primary brain microvascular endothelial cells (BMEC) (**b**) by qPCR. Data were normalized to control cells cultured in standard conditions (normox) which was set as 1 and is presented as an average of three independent experiments with standard deviations. **c** MiR-212/132 levels were measured in mouse cerebral capillaries isolated from mouse brains after traumatic brain injury (TBI) and normalized to expression levels in non-injured hemisphere (control) (*n* = 6/group), *p < 0.05

**Fig. 2:**
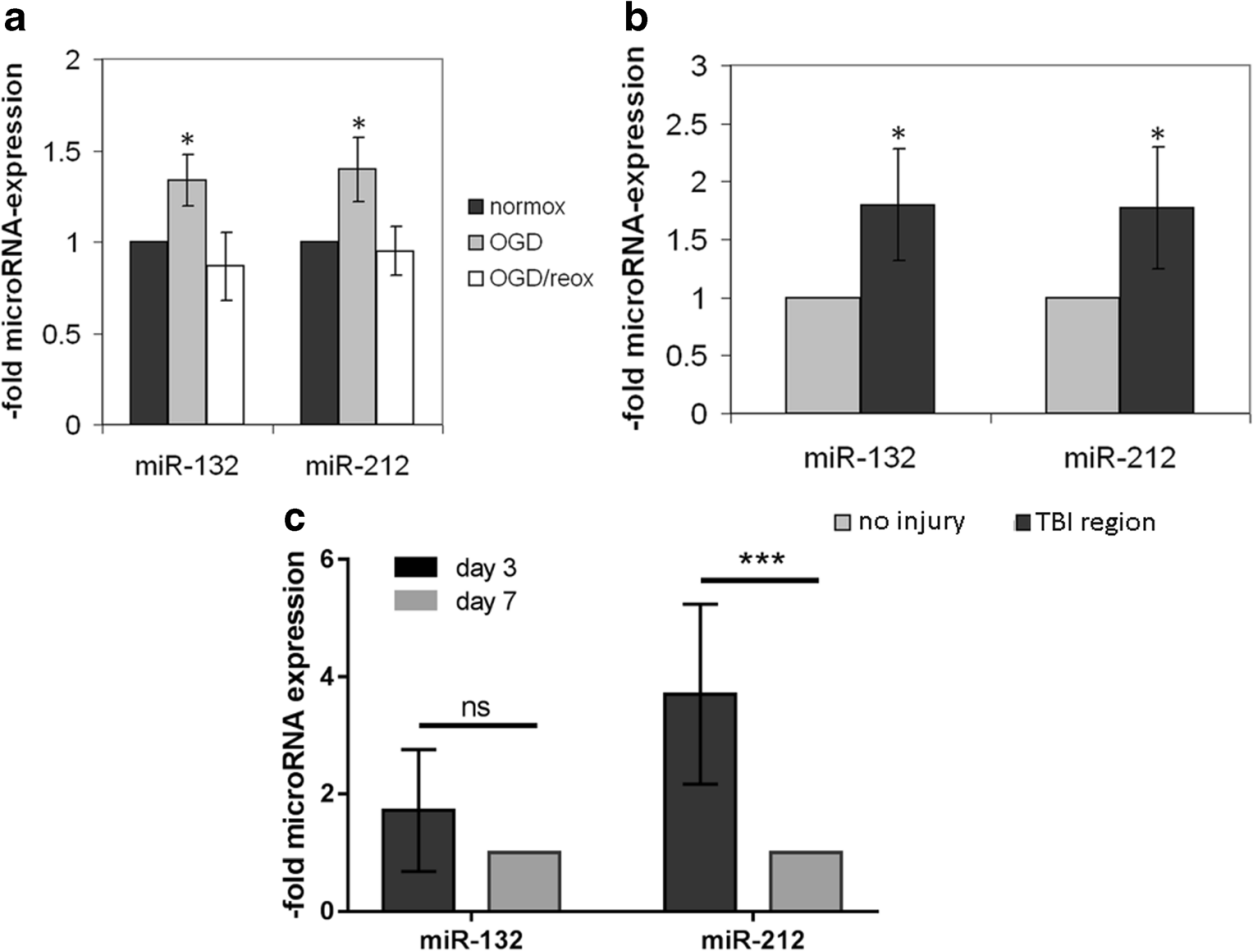
MiR-212/132 are upregulated following hypoxia in human brain microvascular endothelial cells, brain capillaries, and serum exosomes. MiR-212/132 levels were measured in oxygen-glucose deprivation (OGD)- or OGD/re-oxygenation (OGD/reox)-treated human brain microvascular endothelial hCMEC/D3 cell line (**a**) by qPCR. Data were normalized to control cells cultured in standard conditions (normox) which was set as 1 and is presented as an average of three independent experiments with standard deviations. **b** MiR-212/132 levels were tested by qPCR in human brain capillaries isolated post mortem from individuals who died from traumatic brain injury (TBI) and normalized to expression levels in a non-injured brain region (*n* = 5). **c** MiR-212/132 levels were estimated by qPCR in exosomes isolated from patients who suffered from malignant hemispheric stroke. MiR levels measured in samples collected at day 3 post stroke were normalized to levels at day 7 post stroke. Values are presented as mean with standard deviation (*n* = 14 per group), *p < 0.05, ***p < 0.001

The miR-212/132 expression level was measured in the mouse in vitro BBB model, cEND cells, as well as in primary mouse BMEC subjected to 4 h-OGD with or without 20-h re-oxygenation (Fig. 1a, b). CEND cells showed 3- to 10-fold induction of miR-212/132 directly after OGD. The increased expression was also present after 20 h re-oxygenation time (Fig. 1a). Treatment with OGD also led to increased miR-212/132 levels in primary mouse BMEC. However, the induction of miR-212/132 expression was lower than in cEND and was not any more visible after re-oxygenation (Fig. 1b). To confirm these effects in vivo, we isolated capillaries from mouse brains after TBI (Fig. 1c). As a control, the contralateral, non-injured hemisphere of the same brain was used. We observed a highly increased expression of both miR-132 and miR-212 in the injured mouse brain. To ensure that similar hypoxia-induced regulation of miR-212/132 is present in humans, we performed miR-212/132 expression analysis in human BMEC and serum exosomes (Fig. 2). First, we analyzed the human in vitro BBB model, hCMEC/D3 cells after treatment with 4-h OGD and 20-h re-oxygenation (Fig. 2a). MiR-212/132 were significantly increased in hCMEC/D3; however, the observed induction was not present any more after re-oxygenation (Fig. 2a). In cooperation with Forensic Medicine at the University of Würzburg, we isolated capillaries from brains of individuals who died from TBI and suffered from brain hypoxia for several hours before death (Fig. 2b and Table 1). Non-injured brain tissue from the same donor was used as a control, respectively. We detected 2-fold induction of miR-132 and miR-212 in capillaries from the injured brain regions. Further, we analyzed the miR-212/132 expression in serum exosomes from 14 patients with MHS (Fig. 2c). The level of miR-212 was significantly higher at day 3 post stroke as compared to day 7 indicating that this miR is essentially induced in the acute phase of stroke. We could thus confirm the OGD-mediated induction of miR-212/132 in the human BMEC cell line, human brain tissue as well as in serum exosomes of stroke patients.

**Table 1:** Human brain tissue donors for isolation of capillaries and analysis of miR-212/132 expression

Sample	Survival time after injury	Cause of death
1	20 h	TBI
2	7 h	TBI
3	3 h	TBI
4	3 h	TBI
5	0 h	TBI

*TBI* traumatic brain injury

### MiR-212/132 Can Be Detected in Brain Capillaries and Their Enforced Expression Leads to Decrease in Barrier Properties of Endothelial Monolayer

**Fig. 3:**
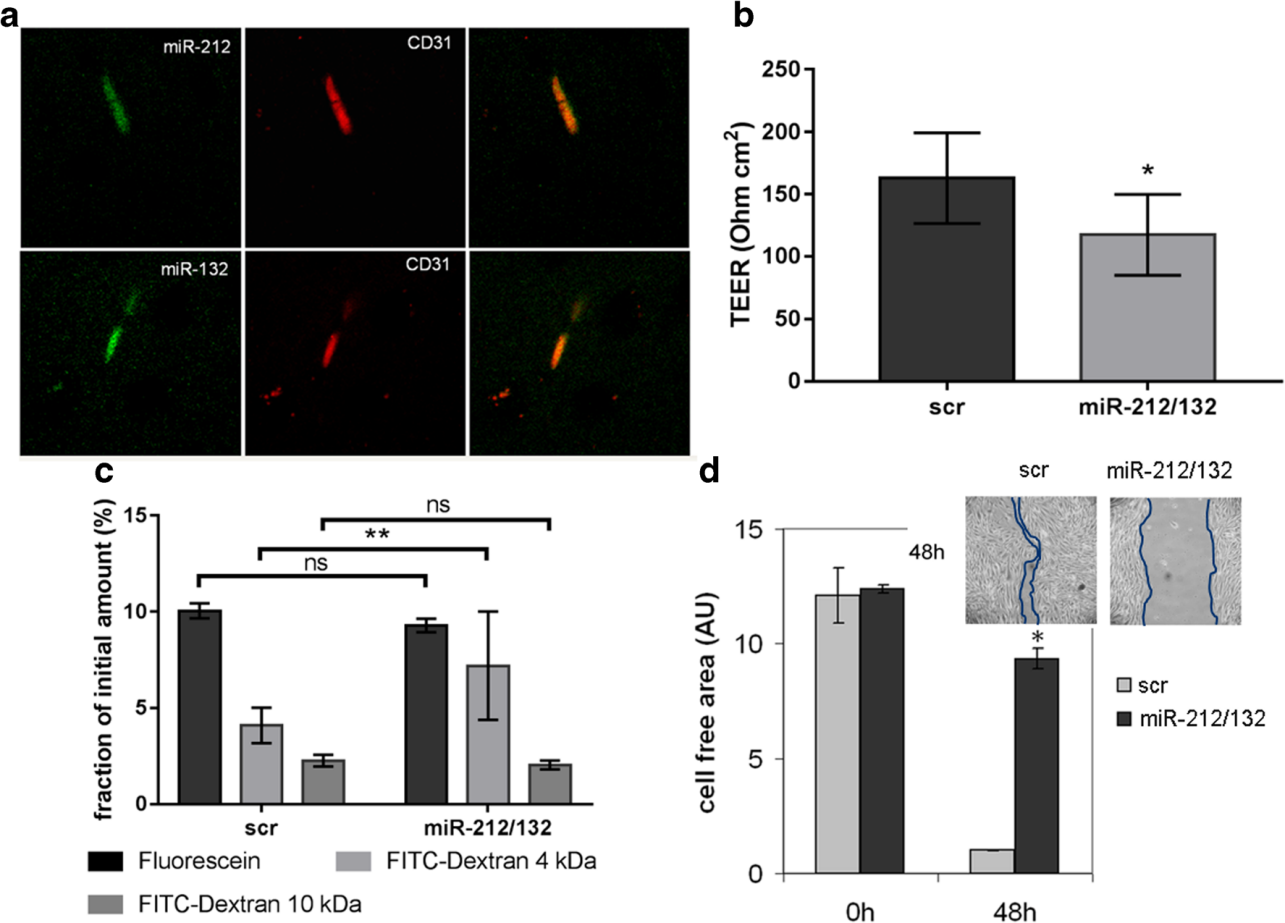
MiR-212/132 can be detected in brain capillaries and their over-expression leads to decrease in barrier properties of endothelial monolayer. MiR-212/132 were detected by in situ hybridization using a specific miR-132 and miR-212 probe. CD31, a marker for endothelial cells, was detected by immunofluorescence (**a**). CEND cells transfected with control (scr) or pre-miR-212/132 (miR-212/132) showed decreased barrier properties as measured by transendothelial electrical resistance (TEER) (**b**) and permeability of fluorescein (332 Da), 4 kDa and 10 kDa FITC-Dextran (**c**). **d** The migration rate of pre-miR-212/132 transfected cEND was measured in the wound healing assay. Images of the migration area were captured 0 and 24 h after removal of the separating chamber. Cell-free area was quantified and presented in arbitrary units (AU). Data are presented as an average of three independent experiments with standard deviations, *p < 0.05, **p < 0.01

In order to demonstrate the brain vascular localization of miR-212/132, we performed an in situ hybridization on mouse brain sections using miR-132 and miR-212 fluorescently labeled probes, respectively. CD31 staining has been used to detect endothelial cells (Fig. 3a). Co-localization of the miR-212/132-probe signal with CD31 staining of brain capillaries was observed, indicating a cerebro-vascular expression of miR-132 and miR-212 (Fig. 3a). In order to detect the phenotypic changes in endothelial cells over-expressing miR-212/132, we transiently transfected cEND cells with pre-miR-212/132, measured TEER values (Fig. 3b) and permeability to fluorescein (332 Da), FITC-Dextran 4 kDa, and FITC-Dextran 10 kDa (Fig. 3c) in comparison to cells transfected with a scrambled (scr) control miR. Over-expression of pre-miR-212/132 in cEND decreased barrier properties of the endothelial monolayer leading to a decreased TEER and increased permeability to FITC-Dextran 4 kDa (Fig. 3b, c). There were no differences detected in the permeability of fluorescein and FITC-Dextran 10 kDa. The process of neovascularization is a known response to hypoxia in the brain [29]. The ability of endothelial cells to migrate can be measured in a wound healing assay. We compared therefore the wound-healing ability of cEND over-expressing pre-miR-212/132 in comparison to cEND transfected with control scrambled miR (Fig. 3d). CEND over-expressing miR-212/132 migrated much slower than the control cells showing a significantly larger cell-free area in the wound healing assay (Fig. 3d).

### Tight Junction and Tight Junction-Associated Proteins Are Direct Targets of miR-132 and miR-212 in Brain Vasculature

**Fig. 4:**
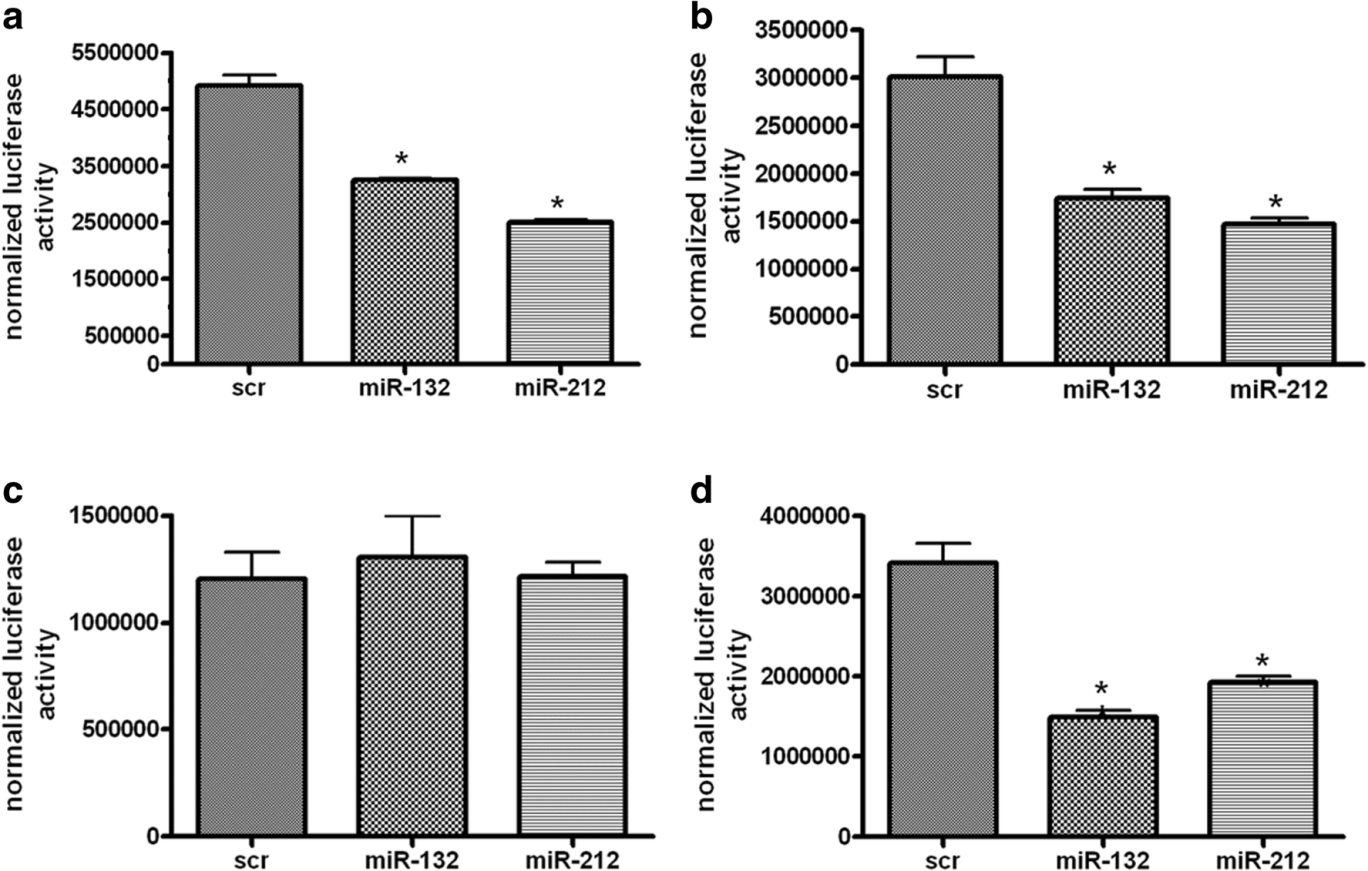
Tight junction and tight junction-associated proteins are direct targets of miR-132 and miR-212. Luciferase activity levels were measured upon co-transfection of a luciferase construct containing 3′-UTR of claudin-1 (Cldn1) (**a**), junctional adhesion molecule 3 (Jam3) (**b**), occludin (Ocln) (**c**), or tight junction-associated protein 1 (Tjap1) (**d**), respectively, with pre-miR-132 or pre-mir-212. Data are presented as an average of three independent experiments with standard deviations, *p < 0.05

MicroRNAs exert their inhibitory actions on mRNA by binding to 3′-UTR of their target genes. Multiple direct targets of miR-212/132 family have been described in the literature [10]. Based on these facts, we selected several predicted/previously validated miR-212/132 targets, such as Bcl2/adenovirus E1B 19 kDa-interacting protein (Bnip2), E1A Binding Protein P300 (Ep300), Matrix metallopeptidase (Mmp9), and Polyadenylate-Binding Protein- Interacting Protein 2 (Paip2) and analyzed their mRNA expression in hypoxic cEND cells (Supplemental Fig. 1) [30–33]. All analyzed targets showed changes in their mRNA expression due to hypoxia and glucose deprivation, either directly after 4-h OGD or after re-oxygenation, which is in accordance with elevated levels of miR-212/132 in hypoxic cEND. We further searched for new targets of miR-212/132 within genes, which could potentially be responsible for the decreased barrier properties of BMEC as presented in Fig. 3b–d. Using bioinformatic analysis by Miranda database as well as experimental data sets of microarray data from cells over-expressing miR-212/132 [11], we selected four predicted targets of miR-212/132 encoding for tight junction proteins, claudin-1 (Cldn1), junctional adhesion molecule 3 (Jam3), occludin (Ocln), and tight junction-associated protein 1 (Tjap1) for further analysis. We tested this bioinformatic prediction using a luciferase reporter assay approach. For this, we cloned the 3′-UTR of Cldn1, Jam3, Ocln, and Tjap1 encompassing miR binding sites downstream of the firefly *luciferase* gene, respectively (Fig. 4a–d). We found that the normalized luciferase activity was significantly reduced upon co-transfection with either pre-miR-132 or pre-miR-212 for Cldn1, Jam3 and Tjap1 (Fig. 4a, b, d). However, no reduction in luciferase activity could be observed for Ocln (Fig. 4c). These results indicate that Cldn1, Jam3, and Tjap1 are direct targets of both miR-132 and miR-212, while Ocln could not be confirmed as a direct miR-212/132 target.

**Fig. 5:**
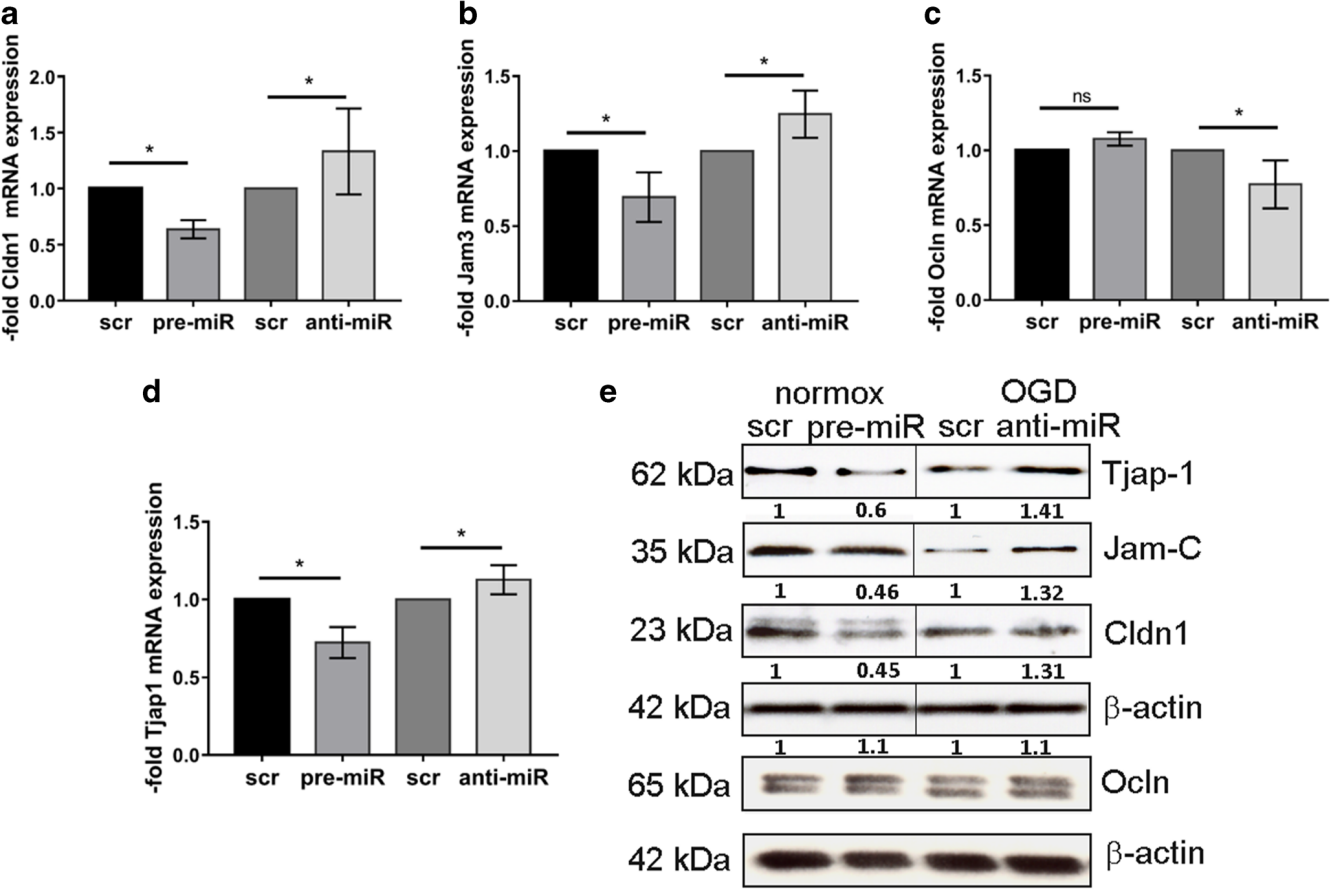
Effects of over-expression of miR-212/132 precursors (pre-) and inhibitors (anti-) on mRNA and protein expression of target genes in cEND cells as compared to the effects of scrambled (scr) controls. CEND were transfected with pre-miR-212/132 or anti-miR-212/132. CEND transfected with anti-miR-212/132 were subjected to 4-h OGD. Analysis of mRNA expression of claudin-1 (Cldn1) (**a**), junctional adhesion molecule 3 (Jam3) (**b**), occludin (Ocln) (**c**), and tight junction-associated protein 1 (Tjap1) (**d**) was performed by qPCR. **e** Protein levels of Claudin-1, Jam-C, Ocln, and Tjap1 in normoxic cEND (normox) over-expressing pre-miR-212/132 and in hypoxic cEND (OGD) transfected with anti-miR-212/132. Representative images were chosen. Numbers under the respective bands show protein levels normalized to β-actin and to scrambled (scr) control. Data are presented as an average of three independent experiments with standard deviations, *p < 0.05

To demonstrate miR-212/132-mediated regulation of Cldn1, Jam3, and Tjap1 in cEND, we transiently transfected cEND cells with pre-miR-212/132 and analyzed mRNA and protein expression (Fig. 5). As expected, mRNA levels of Cldn1 (Fig. 5a), Jam3 (Fig. 5b), and Tjap1 (Fig. 5d) were decreased in cEND over-expressing pre-miR-212/132. No decrease was observed for occludin mRNA (Fig. 5c). Accordingly, protein levels of claudin-1, Jam-C, and Tjap1 were decreased in cEND over-expressing pre-miR-212/132 (Fig. 5e). MiR-132 and -212 are induced in OGD but their action can be inhibited by anti-miRs. Thus, we transfected cEND cells with anti-miR-212/132 and subjected the cells 24 h later to 4-h OGD. The anti-miR-212/132 transfection could reconstitute the expression of Cldn1 (Fig. 5a), Jam3 (Fig. 5b), and Tjap1 (Fig. 5d) mRNA in hypoxic cEND. This effect was not observed for occludin (Fig. 5c). Similarly, cEND transfected with anti-miR-212/132 showed higher protein levels of claudin-1, Jam-C, and Tjap1 (Fig. 5e) than cells transfected with scrambled (scr) control. No changes in occludin protein level could be observed (Fig. 5e). These results indicate that the level of miR-212/132 targets can be influenced in BMEC either by over-expression/induction of miR-212/132 or by the miR-212/132 inhibition, respectively.

## Discussion

The goal of this study was to determine the impact of miR-212/132 on BBB properties under hypoxic conditions. We identified three miR-212/132 targets with documented roles in the regulation of barrier properties at the BBB. This can provide a potential mechanism of BBB damage under oxygen-glucose deprivation.

Epithelial and endothelial cell-cell contacts play an important role in the protection of organ homeostasis and they are often being dysregulated in diseases, such as cancer, ischemic and/or hypoxic disease states, and infections [1, 27, 34–36]. Stroke and TBI represent the second most frequent causes of death and disability with limited therapeutic options [37]. Multiple changes at the BBB have been reported in stroke and TBI, such as decreased TJ protein expression, extended transendothelial permeability, and inflammation [2, 38]. MiRs have been reported to regulate multiple tight junction proteins and to modulate epithelial/endothelial barrier functions [39]. In this report, we show that miR-212/132 is highly induced in hypoxic BMEC. We showed an increase of miR-212/132 in hypoxic immortalized mouse BMEC, as well as in primary mouse BMEC and in capillaries isolated form mouse brains after TBI. We hypothesized that a similar regulation of miR-212/132 might also take place in human BMEC. Consistent with this hypothesis, miR-212/132 were upregulated in the hypoxic human in vitro BBB model, in human post mortem TBI samples as well as in serum exosomes from patients with MHS. Our results are in line with other reports showing hypoxia-induced expression of miR-132 and/or miR-212 in isolated hippocampal neurons, in a model of hindlimb ischemia, and in Schwann cells [40–43]. To the authors’ best knowledge, this is the first report showing OGD-mediated induction of miR-212/132 in BMEC.

In order to elucidate the cellular mechanisms mediated by hypoxia-induced miR-212/132, we searched for miR-212/132 direct targets within TJ proteins. While an increase in TJ protein expression is typically related to lower permeability and neuroprotection, low TJ protein expression accompanies multiple brain disorders with compromised barrier properties [44]. For instance, over-expression of miR-212 led to a hyperpermeability of CaCo2 monolayers and resulted in decreased expression of the TJ protein ZO-1 [45]. Claudin-1 and Jam-C, which we identified as direct miR-212/132 targets, are components of TJ complex. Claudin-1 belongs to a group of sealing claudins. It seals TJs in skin epithelial and lung endothelial cells as well as in the choroid plexus epithelium [46–48]. The expression of claudin-1 in brain capillaries ameliorated chronic experimental autoimmune encephalomyelitis and reduced BBB leakiness for blood borne tracers in mice [49]. The presence of claudin-1 at the BBB is still a matter of debate due to the use of a claudin-3 cross-reactive antibody in several reports [50]. However, in the cEND in vitro BBB model, we detected claudin-1 expression using qPCR and Western blotting with a specific antibody. In accordance with that, claudin-1 could be detected in other in vitro BBB models, such as hCMEC/D3 and HBMEC [21, 51]. Jam-C is a widely expressed adhesion molecule localized in BMEC at the TJ. Jam-C knockout mice were reported to develop a severe hydrocephalus [52]. Interestingly, a loss of function mutation in the Jam3 gene has been found in humans. The deficiency of Jam-C in humans leads to hemorrhagic destruction of the brain, subependymal calcification, and congenital cataracts indicating multiple roles of Jam-C in the CNS [53]. In this report, a direct regulation by miR-212/132 resulted in a lower Jam-C expression, which could be rescued when miR-212/132 were specifically inhibited. Jam-C is known to play a role in the regulation of endothelial permeability, i.e., disruption of Jam-C function led to a decreased permeability in VEGF-stimulated dermal microvascular endothelial cells [54]. Another miR-212/132 target is Tjap1 (also known as Pilt). Tjap1 has been previously validated as a direct target of miR-212/132 [31, 32]. Herein, we show a miR-212/132-mediated regulation of Tjap1 in the brain vascular endothelium. Tjap1 is localized toward the cytoplasmic face of TJs and within a Golgi stack [55]. The physiological role of Tjap1 at the BBB has not been elucidated yet. Due to its localization at the TJs we thought it might contribute to regulation of endothelial permeability. We showed that a direct binding of either over-expressed or hypoxia-induced miR-212/132 led to a downregulation of Cldn1, Jam-C, and Tjap1 in BMEC. This resulted in lower TEER values, raised permeability to FITC-Dextran 4 kDa as well as in slower migration of BMEC as observed in the wound healing assay. Compromised barrier properties in BMEC over-expressing miR-212/132 indicate that an increase of miR-212/132 alone is sufficient to promote BBB damage. Since miR-212/132 regulate multiple targets in vascular endothelium [11], additive effects of this regulation might be expected.

Due to the fact that miR-212/132 knockout pups at postnatal day 5 exhibit a dramatic increase in retinal vasculature development in comparison to wild-type mice, the miR-212/132 cluster has been proposed to have general anti-angiogenic properties [11]. Particularly, the miR-212 plays a role in the regulation of endothelial function. In other cell types, such as skin fibroblasts, miR-132 was shown to promote migration, while migration and invasion of cancer cells could be inhibited by miR-212/132 [56, 57]. Moreover, in an in vivo model of hindlimb ischemia, miR-212/132 was demonstrated to enhance arteriogenesis through modulation of the Ras-MAPK pathway via directly targeting its inhibitors Rasa1 and Spred1 [41]. Similar findings were gained in a tumor angiogenesis model for miR-132 and its direct target p120RasGAP [58]. Thus, depending on the type of tissue, miR-212/132 is able to increase or inhibit cellular migration. The inhibitory effect of miR-212/132 over-expression on endothelial migration presented here is further in line with enhanced retinal angiogenesis in miR-212/132 knockout mice [11].

In summary, this study demonstrates that the miR-212/132 family contribute to compromised barrier properties in hypoxic BMEC via a mechanism involving the regulation of Cldn1, Jam-C, and Tjap1. However, to elucidate the precise signaling pathway involved in this phenomenon and to use it as a therapeutic option in hypoxic brain disorders, further investigation is required.

## Electronic Supplementary Material

ESM 1 (PDF 253 kb)
