# Supplementary material for: Hypoxia-Induced MicroRNA-212/132 Alter Blood-Brain Barrier Integrity Through Inhibition of Tight Junction-Associated Proteins in Human and Mouse Brain Microvascular Endothelial Cells
Source: Transl Stroke Res. 2019 Jan 8;10(6):672–83. doi: 10.1007/s12975-018-0683-2 (PMC6842347; doi:10.1007/s12975-018-0683-2)
Supplement: Supplementary file 1 — (PDF 253 kb) [file 12975_2018_683_MOESM1_ESM.pdf]

Hypoxia-induced microRNA-212/132 alter blood-brain barrier integrity through inhibition of tight junction-associated proteins in human and mouse brain microvascular endothelial cells

Malgorzata Burek[1*], Anna König[1], Mareike Lang[1], Jan Fiedler[2], Sabrina Oerter[3], Norbert Roewer[1], Michael Bohnert[3], Serge C. Thal[4], Kinga G. Blecharz-Lang[5], Johannes Woitzik[6], Thomas Thum[2], Carola Y. Förster[1]

[1] Department of Anesthesiology and Critical Care, University of Würzburg, Würzburg, Germany

[2] Hannover Medical School, Institute of Molecular and Translational Therapeutic Strategies (IMTTS), Hannover, Germany

[3] Institute of Forensic Medicine, University of Würzburg, Würzburg, Germany

[4] Department of Anesthesiology, Medical Center of the Johannes Gutenberg-University Mainz, Mainz, Germany

[5] Department of Experimental Neurosurgery, Charité – Universitätsmedizin Berlin, Corporate Member of Freie Universität Berlin, Humboldt-Universität zu Berlin and Berlin Institute of Health, Berlin, Germany

[6] Department of Neurosurgery and Center for Stroke Research Berlin (CSB), Charité - Universitätsmedizin Berlin, Berlin, Germany

*Corresponding author:Malgorzata Burek, PhD, Universitätsklinikum Würzburg, Klinik und Poliklinik für Anästhesiologie, Oberdürrbacher Straße 6, 97080 Würzburg, Germany; Phone: +4993120130046; Fax: +4993120130019, E-mail: Burek_M@ukw.de

SUPPLEMENTAL INFORMATION_ Burek *et al.*

Supplemental Figure 1

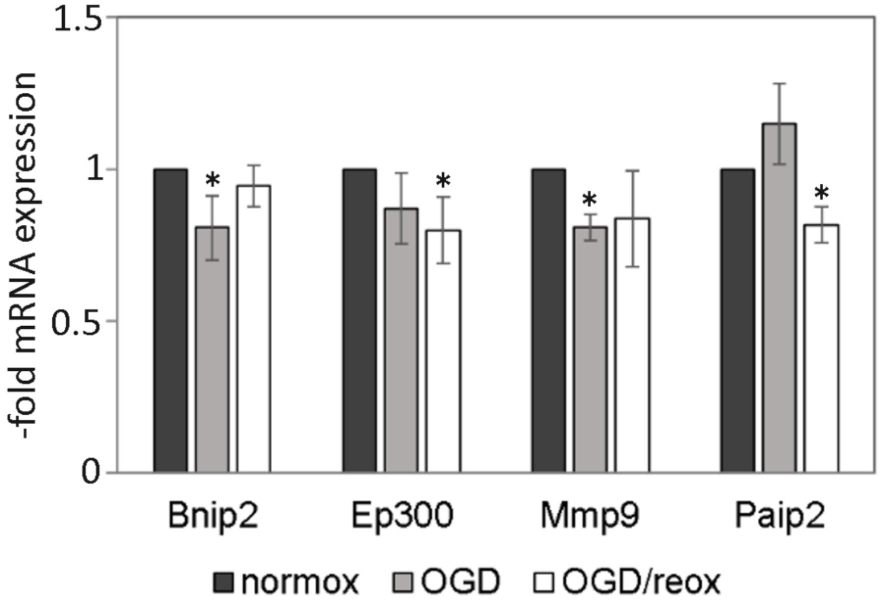

Supplemental Figure 1. Expression of predicted / validated miR-212/132 targets in hypoxic cEND cells. mRNA expression of Bcl2/adenovirus E1B 19 kDa-interacting protein (Bnip2), E1A Binding Protein P300 (Ep300), Matrix metallopeptidase (Mmp9) and Polyadenylate-Binding Protein- Interacting Protein 2 (Paip2), all predicted / validated targets of miR-212/132 was analyzed in cEND subjected to 4-hour-OGD (OGD) with or without 20-hour-re-oxygenation (OGD/reox) by qPCR. Data are presented as average of three independent experiments with standard deviations, * $p < 0.05$.
